# Molecular Characterization of the miR156/MsSPL Model in Regulating the Compound Leaf Development and Abiotic Stress Response in Alfalfa

**DOI:** 10.3390/genes13020331

**Published:** 2022-02-10

**Authors:** Xueyang Min, Kai Luo, Wenxian Liu, Keyou Zhou, Junyi Li, Zhenwu Wei

**Affiliations:** 1 College of Animal Science and Technology, Yangzhou University, Yangzhou 225009, China; keuchow@foxmail.com (K.Z.); zwwei@yzu.edu.cn (Z.W.); 2 Key Laboratory of Sustainable Utilization of Tropical Biological Resources of Hainan Province, School of Tropical Crops, Hainan University, Haikou 570228, China; luok@hainanu.edu.cn (K.L.); ljy928688582@163.com (J.L.); 3 State Key Laboratory of Grassland Agro-Ecosystems, College of Pastoral Agriculture Science and Technology, Lanzhou University, Lanzhou 730000, China; liuwx@lzu.edu.cn

**Keywords:** *Medicago sativa*, SPL transcription factor, leaflet number, compound leaf, abiotic stress

## Abstract

Plant leaf patterns and shapes are spectacularly diverse. Changing the complexity of leaflet numbers is a valuable approach to increase its nutrition and photosynthesis. Alfalfa (*Medicago sativa*) is the most important forage legume species and has diversified compound leaf patterns, which makes it a model species for studying compound leaf development. However, transcriptomic information from alfalfa remains limited. In this study, RNA-Seq technology was used to identify 3746 differentially expressed genes (DEGs) between multifoliate and trifoliate alfalfa. Through an analysis of annotation information and expression data, *SPL*, one of the key regulators in modifiable plant development and abiotic stress response, was further analyzed. Here, thirty *MsSPL* genes were obtained from the alfalfa genome, of which 16 had the putative miR156 binding site. A tissue expression pattern analysis showed that the miR156-targeted *MsSPLs* were divided into two classes, namely, either tissue-specific or widely expressed in all tissues. All miR156-targeted *SPL*s strongly showed diversification and positive roles under drought and salt conditions. Importantly, miR156/MsSPL08 was significantly suppressed in multifoliate alfalfa. Furthermore, in the paralogous mutant of *MsSPL08* isolated from *Medicago truncatula,* the phenotypes of mutant plants reveal that *miR156/MsSPL08* is involved not only involved the branches but also especially regulates the number of leaflets. The legume is a typical compound leaf plant; the ratio of the leaflet often affects the quality of the forage. This study sheds light on new functions of *SPL* genes that regulate leaflet number development.

## 1. Introduction

Each step of biological and developmental progress requires appropriate transformation and expression changes in plants, such as the formation of organs, the division of cells, and the production of metabolites [1,2]. Leaves are the major component of plant architecture, and the main tissue of photosynthesis, thermoregulation, and gas exchange. Plant leaf forms are spectacularly diverse, and can be grouped into simple leaves and compound leaves. Compound leaves exhibit more diversity than simple leaves, particularly because of a specific morphogenetic process [3]. Alfalfa is the most widely cultivated legume for livestock, with leaves having a typical trifoliate pattern [4]. As one of the forages with the highest digestible protein, 70% of the protein is stored in the leaves, and the cellulose content in the leaves is only 1/3 of the stem [5]. Therefore, studying the molecular mechanism of alfalfa multifoliate formation can provide a strong theoretical basis for creating new alfalfa germplasm resources with high protein content.

Generally, the class 1 *KNOTTED-LIKE HOMEOBOX* (*KNOX1*) is recognized as a conserved pathway that regulates plant compound leaf differentiation. Additionally, PALM1 (Cys(2)His(2) zinc finger transcription factor) regulates the spatial-temporal expression of *SINGLE LEAFLET1* (*SGL1*), which controls leaflet formation, in *Medicago truncatula*. CRISPR/Cas9 genome editing suggested that *MsPALM1* regulates multifoliate formation in alfalfa [6]. A recent study found a new mechanism for regulating compound leaf patterning in which PINNATE-LIKE PENTAFOLIATA1 (PINNA1) is a determining factor of morphogenetic activity in inverted repeat-lacking clade (IRLC) legume plants [7]. Considering the importance of the complexity of the leaves to the quality of forage plants, further studies are required to explore the precise control mechanisms.

Transcription factors (TFs) are DNA-binding proteins that coordinate precise gene expression, which gives rise to distinct phenotypic outputs by activating and/or repressing the transcription of multiple target genes [8,9]. Among many types kinds of TF families, *SPL* encodes a special family of TFs that are unique to plants. *SPL* TFs are defined by containing 76 highly conserved amino acid residues, including two Zn-finger-like structures (Cys-CysHis-Cys and Cys-Cys-Cys-His), and a nuclear localization signal (NLS) motif [8,10]. To date, *SPL* genes have been recognized in various plant species, which range from the unicellular algae [11], *Physcomitrella patens* to the highest number, 177 *SPL* genes [12], identified in cotton [13]. Current studies in higher plants have suggested that *SPL* genes control diverse aspects of plant development and stimulus responses, including flowering time, fruit development and ripening, root development, vegetative phase transition, leaf initiation rate, trichome distribution on the stem, floral organ development, fertility, and stress responses [2,14,15,16,17,18,19]. In many developmental processes, the role of some *SPL* genes is post-transcriptionally regulated by miR156 (microRNAs156), as their transcripts carry functional response elements to these highly related miRNAs. All *Arabidopsis thaliana SPL* miRNA-responsive elements (MREs) are downstream of the SBP-domain and located in a part of the last exon, except for *SPL3*, *SPL4*, and *SPL5*, which lie in the 3′-untranslated region (3′-UTR), and all of them promote flowering time and vegetative phase transition [2,20,21]. In *Arabidopsis*, miR156 acts by repressing the expression of 10 *SPL* genes, which results in gain-of-function and loss-of-function phenotypes that divide the genes into the following three functionally distinct groups: the vegetative phase transition (*SPL2*, *SPL9-11*, *SPL13,* and *SPL15*); the floral meristem identity transition (*SPL3-4* and *SPL5*), and certain physiological processes (*SPL16*) [22]. Si et al. tested the function of *OsSPL13*, which is recognized as a quantitative trait locus. A functional analysis showed that *OsSPL13* enhanced rice grain length and yield, and bioinformatics analysis results suggested that it has an OsmiR156 complementary site located in the 3′ UTR [23]. The ideal plant architecture 1 (*IPA1*) quantitative trait locus encodes *OsSPL14* and is regulated by OsmiR156 in rice. A functional analysis indicated that the *OsSPL14* gene may help increase rice grain yield by increasing lodging resistance, enhancing grain yield per panicle, and reducing unproductive tillers [24]. Although the amount of functional and regulatory data on *SPL* genes is increasing, the physiological and biological characteristics of many members still need further clarification.

Recently, a chromosome-level genome assembly has been generated for cultivated alfalfa, which offers the capability of comprehensively analyzing the *SPL* gene family in this species. However, an overall analysis of *MsSPL* genes has not been performed in alfalfa, except by Ruimin Gao, who found seven *SPLs* regulated by miR156 in alfalfa through a next-generation RNA sequencing method [25]. Given the importance of *SPL* genes in the regulation of plant traits and abiotic stress adaptation, we systematically evaluated the *SPL* genes at the genome-wide level by using a rigorous method. Furthermore, *M. truncatula* was used as a model to verify the function of *MsSPL08* in regulating multifoliate leaf development. The results provide valuable information for accelerating *SPL* gene research in this important forage crop.

## 2. Materials and Methods

### 2.1. Plant Material and Abiotic Stress Treatment

Two first-generation backcross populations were constructed from a cross between multifoliate alfalfa “PL34HQ”, and trifoliate alfalfa “Huaiyin”. A single F_1_ plant from the cross was used as the female parent, trifoliate alfalfa “Huaiyin” as a recurrent parent to create the BC_1_ backcross populations. Then choose the offspring with multifoliate trait as the female parent, “Huaiyin” alfalfa as a father to create the BC_2_ backcross populations. Finally, we obtained two genetic strains: multifoliate (pinnate pentafoliate leaves in most cases) and trifoliate alfalfa, which were used as RNA sequencing materials. The leaflets were collected from the apical meristem of five vegetative growth stage plants in May 2019 (active stage of physiological activity), then mix them as one biological replicate. Three biological replicates were applied for each leaf type, all sampled leaflets were rizol Kit (Invitrogen, Gaithersburg, MD, USA) immediately frozen in liquid nitrogen and stored at −80 °C for RNA isolation. A *M. truncatula mtspl* mutant (ecotype R108: NF10482-4) was isolated from a tobacco (*Nicotiana tabacum*) *Tnt1* retrotransposon-tagged mutant collection by forward genetic screening [26]. The MtSPL4 gene has one exon with 423 nucleotides. The *MtSPL4* gene was tested using the following primers designed based on MtSPL4: NF10482_F: TTCACCTGTGTTCTTGTGCAT, NF10482_R: TGGGACATTTGATCACTACTCTT, LTR6-R: GCTACCAACCAAACCAAGTCAA.

Alfalfa seeds (cultivar Gannong No. 9) were sterilized in 1.0% (*v*/*v*) sodium hypochlorite and germinated at 25 °C for 5 days. Then the uniform seedlings were cultivated into the sand culture at 25 °C, 50% relative humidity, and 16-h light/8-h dark conditions. After 30 days of cultivating, the uniform growth seedlings were classified into two groups: one was keeping the culture conditions as control, the other one was subjected to 20% PEG-6000 and 300 mM NaCl for 1, 2, and 4 days. Three biological replicates were applied for each time point. After treatment, the young root was immediately frozen in liquid nitrogen and stored at −80 °C for RNA isolation.

### 2.2. RNA Isolation, Illumina Library Construction, and Sequencing

Total RNA from each sample was separately isolated for Trizol Kit (Invitrogen, Gaithersburg, MD, USA). In total, six RNA samples were obtained, representing the two leaf types from five single plants. RNA was quantified and quality-checked for each sample using one percent of agarose gels and Agilent 2100 Bioanalyzer (Agilent Technologies, Santa Clara, CA, USA). Furthermore, the cDNA libraries were constructed according to the manufacturer’s instructions of the NEBNext UltraTM RNA Library Prep Kit for Illumina (NEB, Shanghai, China). The cDNA library was sequenced using the HiseqXTen platform at Novogene (Beijing, China). FastQC raw data were processed using Trimmomatic (Version 0.39) to obtain clean reads by removing reads containing N base and adapter, low-quality bases from reads 3′ to 5′and 5′ to 3′ (Q value < 20), and the bases whose tail mass value is less than 20 (the window size is 5 bp).

### 2.3. Bioinformatics Analysis of RNA-Seq Data

The genome assembly and annotation files (Version 3) of alfalfa from the figshare were used as the reference [6]. The TPM (Transcripts Per Million) method was used to calculate expression patterns through StringTie software (version 1.3.3b). Differentially expressed genes (DEGs) were conducted using the DESeq2 R package, and the *q*-Value < 0.05, FoldChange > 2 were defined as DEGs. The DEGs Gene Ontology (GO) and the Kyoto Encyclopedia (KEGG) enrichment analyses were implemented using the clusterProfiler (*q*-Value < 0.05). The software of HISAT2 was used to map the clean reads onto the cultivated alfalfa (cultivar XinJiangDaYe) genome.

### 2.4. Identification of Alfalfa SPL Genes

Alfalfa genome data were derived from the Figshare website (https://figshare.com/articles/dataset/genome_fasta_sequence_and_annotation_files/12327602, accessed on 9 July 2021). Initially, the conserved SBP domain (PF03110) based on Hidden Markov Model (HMM) was retrieved from the PFAM database (http://pfam.xfam.org/, accessed on 9 July 2021) and was employed to search the alfalfa protein sequence with the threshold of e  <  1 × 10^−5^. Furthermore, a TBLASTN homology search was performed against the alfalfa genome using *Arabidopsis,* rice, and *M.truncatula* SPL proteins, to identify additional SPLs. For the SPL with splice variants, only the primary one was kept and redundant sequences were removed using the CD-HIT tool (http://weizhongli-lab.org/cd-hit/, accessed on 9 July 2021). The protein length, molecular weight, grand average of hydropathy (GRAVY), and theoretical isoelectric point (*pI*) of each MsSPL protein were calculated within the ExPasy program (http://www.expasy.org/, accessed on 12 July 2021).

### 2.5. SPL Gene Bioinformatics Analysis in Alfalfa

The physical location of the *MsSPL* was displayed to chromosomes using TBtools software. The exon/intron structures of *MsSPL*s were analyzed in the GSDS 2.0 (http://gsds.gao-lab.org/, accessed on 12 July 2021). Conserved motifs of MsSPL proteins were identified using the MEME (http://meme-suite.org/tools/meme, Version 5.2.0) (accessed on 12 July 2021), with the maximum number of the motif is ten. The SPL protein sequences of MtSPLs (24), AtSPLs (16), and OsSPLs (19) were downloaded from the Phytozome 12 database (https://phytozome.jgi.doe.gov/pz/portal.html, accessed on 12 July 2021). Multiple protein sequence alignment was conducted using the ClustalW program. An unrooted phylogenetic tree was constructed with the maximum likelihood method and 1000 bootstrap by MEGA 7.0 software. Collinear blocks were evaluated by MCScan. The syntenic relationship between the *MsSPL* genes and *SPL* genes from *A. thaliana*, *Oryza sativa*, *M. truncatula*, and *Glycine max* were determined by using TBtools [27]. Furthermore, the synonymous substitution rate (*Ks*) and nonsynonymous substitution rate (*Ka*) ratios were calculated by the software of TBtools. The divergence date (T) of collinear gene pairs was estimated using the formula T = Ks/2λ, where λ was 1.5 × 10^−8^ in dicots [28].

### 2.6. Prediction of the miR156 Target Site and Promoter Cis-Element

The *MtmiR156* sequences were obtained from the miRBase (http://www.mirbase.org/, accessed on 20 July 2021). Then the psRNATarget server (http://plantgrn.noble.org/psRNATarget/, accessed on 20 July 2021) was employed to predict potentially targeted by miR156 in alfalfa CDS and 3′ UTRs regions of all *MsSPL*s for complementary sequences, with the maximum expectation of three.

The 2.0 kb upstream sequence of the predicated *MsSPL* genes were used to analyze the putative stress or hormone-responsive *cis*-acting regulatory elements in PlantCARE online database [29].

### 2.7. Expression Analysis of MsSPLs in Different Tissues

The *MsSPL* genes transcriptional data of 6 tissues, including PES (post-elongation stem internodes), flowers, leaf, nitrogen-fixing root nodules, ES (elongating stem internodes), and root, were obtained from LegumeIP V3 (http://plantgrn.noble.org/LegumeIP/gdp/12/gene, accessed on 20 July 2021) to reveal their function in alfalfa growth and development [30].

### 2.8. RNA Isolation and qRT-PCR Verification

Total RNA was isolated using Sangon UNIQ-10 column Trizol total RNA extraction kit (TaKaRa, Dalian, China). cDNA synthesis, qRT-PCR reactions, and data analysis were performed according to previous studies [31], the actin gene used to evaluate the relative fold differences based on the comparative *Ct* method. The gene-specific primers were designed by the Primer3 software (Table S1).

### 2.9. Statistical Analysis

In the initial sampling portion of the entire experiment, we used three independent biological replicates. RNAseq analysis, *SPL* genes identify and bioinformatics analysis described in the Section 2 part. In addition, the significance between control and treatment groups were evaluated using Duncan’s multiple range test in SPSS 20. Graphs were generated with Photoshop 7.

## 3. Results

### 3.1. Illumina Sequencing, Mapping, and Annotation

**Figure 1:**
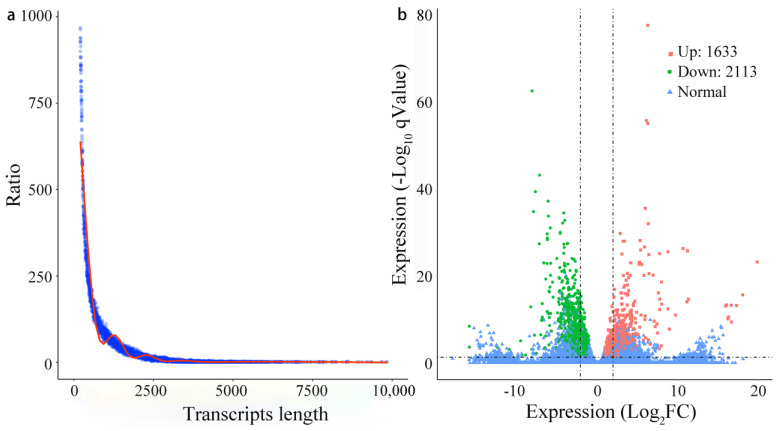
(**a**) Length distribution of the assembled genes. (**b**) Volcano plots display log_2_ converted fold changes and FDR values of the DEGs.

To deeply understand the compound leaf development of alfalfa at the transcriptome level, after filtering out low-quality reads, approximately 0.245 billion clean reads were generated. The GC content of clean data ranged from 43.84% to 45.09%, and the Q30 ranged from 92.70% to 97.19%, with an average of 93.90% from six RNA samples (Table 1). Overall, the average total mapped, multi mapped and uniquely mapped clean read ratios were 91.89%, 58.12%, and 33.77%, respectively (Table 1). In total, 164,632 genes with 251,850 transcripts were detected with a maximum of 13,074 bp, and the mean length was 757.78 bp with an N50 of 1135 kb (Figure 1a). The functional annotation of all genes aligned with six commonly used public databases, which revealed that the number of genes ranged from 88,116 (53.52%, KEGG) to 163,176 (99.12%, NR), and 44,239 (27%) genes were annotated in all six databases (Table S2).

### 3.2. Functional Annotation and Transcription Factor Identification

**Figure 2:**
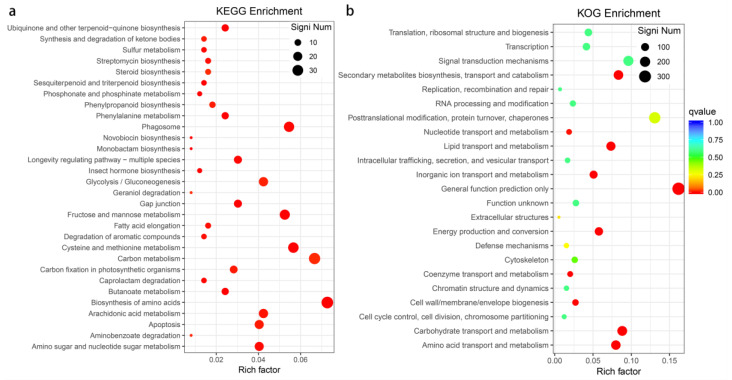
KEGG (**a**) and KOG (**b**) pathway enrichment scatter diagram of DEGs between multifoliate and trifoliate alfalfa. The size and color of the dots represent the gene number and the range of the −log_10_ (*q*-Value), respectively.

Among 3746 DEGs, 1633 DEGs were upregulated and 2113 DEGs were downregulated between multifoliate and trifoliate alfalfa (Figure 1b). A GO classification summarized all DEGs into three main categories including 64 functional groups (Figure S1). In the biological process (BP) category, “rhythmic process” (105) and “response to stimulus” (1130) were highly represented groups. Within the cellular component (CC) category, the “extracellular region” (321) was the most abundant group, followed by “nucleoid” (8). For the molecular function (MF) category, “catalytic activity” (1772), “nutrient reservoir activity” (34), and “transporter activity” (244) were the top 3 highly represented groups. All genes were further assigned to the KEGG and KOG databases. A total of 495 genes were assigned to 184 KEGG pathways, and the top 30 significant enrichment pathways are displayed in Figure 2a. “Fructose and mannose metabolism” (26, 5.25%) was the most significantly enriched pathway, followed by “Cysteine and methionine metabolism” (28, 5.66%), “Biosynthesis of amino acids” (36, 7.27%), and “Ubiquinone and another terpenoid-quinone biosynthesis” (12, 2.42%). Furthermore, 2028 genes were annotated in 23 individual KOG functional classes, with “Amino acid transport and metabolism” (162, 7.99%) being the most significant enrichment term, followed by “Lipid transport and metabolism” (149, 7.35%), “Carbohydrate transport and metabolism” (179, 8.83%), and “Inorganic ion transport and metabolism” (103, 5.08%) (Figure 2b).

TFs are master regulators of gene expression by binding to their promoter regions. A total of 227 DEGs were identified as TFs and classified into 29 gene families (Figure S2). Among the recognized TFs, bHLH has the most members at 19, followed by the ERF (ethylene response factor: 17) (17), NAC (NAM, ATAF, and CUC: 15), MYB_related (V-myb myeloblastosis viral oncogene homologue: 14), and GATA (14) families. Compared with upregulated TFs, most of them were downregulated, such as ERF, DBB (double B-box), and Dof (DNA binding with one finger), which were all downregulated, and NAC and SPL, which had only one and two members, respectively, were upregulated. Among the various TF members, *SPL* genes are post-transcriptionally regulated by miR156 and play roles in plant morphology and development. This study found that the expression of *SPLs* was significantly suppressed in multifoliate alfalfa. Thus, the genome-wide characterization of the *SPL* genes in alfalfa was further analyzed.

### 3.3. Identification of MsSPL Genes in Alfalfa

A total of 30 *SPL* putative genes were identified in alfalfa after removing redundant sequences. Furthermore, a uniform nomenclature was assigned to these genes based on the order of their chromosomal locations (Table 2). Among the 16 *SPL* genes in *Arabidopsis*, AtSPL15 and AtSPL16 no orthologues in alfalfa; AtSPL2 has the maximum orthologues (five); AtSPL13 has four paralogues; AtSPL1, AtSPL6, and AtSPL14 have three paralogues; AtSPL2, AtSPL10, AtSPL11, and AtSPL12 have two paralogues; and remaining genes have one (Table 2). Among the 30 MsSPL proteins, the relative molecular weights varied from 16.58 kDa (MsSPL08) to 113.32 kDa (MsSPL08), and from 141 (MsSPL08) to 1025 (MsSPL05 and MsSPL07) amino acids (aa) in length, with 21 members showing a *pI* > 7 and the remaining showing a *pI* < 7. The GRAVY index of all MsSPLs below zero indicated that all of the deduced polypeptides were hydrophilic.

### 3.4. Phylogenetic, Gene Structure, Conserved Motif, and Domain Analyses

**Figure 3:**
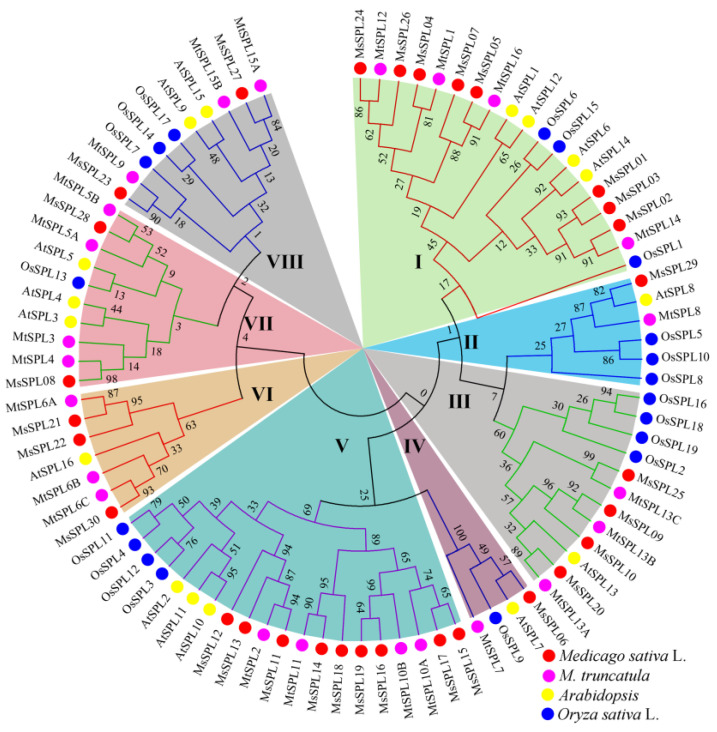
Unrooted phylogenetic tree representing the relationships among *M. sativa*, *Arabidopsis*, *M. truncatula*, and *O.sativa*. The different color background indicates the different subgroups.

To study the evolutionary correlations of MsSPLs, an unrooted phylogenetic tree was created using the full-length SPL proteins of *M. truncatula*, *Arabidopsis*, alfalfa, and rice by the maximum likelihood algorithm in MEGA 7.0, which divided the 30 MsSPL proteins into eight distinct groups (Figure 3). As expected, the SPL proteins from legumes generally exhibited closer relationships, than those from rice (monocot). Except for subfamily VI SPLs from dicots, the remaining subfamilies contained four species. The phylogenetic analysis suggests that the *SPL* genes have the conserved characteristics of dicots and monocots.

**Figure 4:**
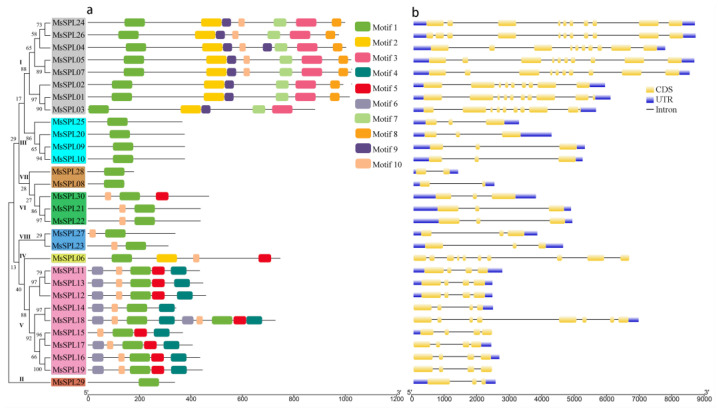
The architectures of the conserved protein motifs (**a**) and gene structures (**b**) of the *SPL* genes in alfalfa.

To examine the diversity in the MsSPLs, 10 conserved motifs were predicted (Figure 4a). The number of conserved motifs in 30 MsSPL proteins varied from 1 to 9. Generally, the MsSPL proteins that shared similar motif compositions tended to cluster together. All MsSPLs contained motif 1, which represented two Zn-finger-like structures, and NLS. In addition, some groups were found to share specific motifs. For instance, motifs 3, 7, 8, and 9 are found exclusively in Group I, and groups II, III, and VII only contain only motif 1.

Gene structural features are known to play a crucial role in the evolution of gene families. We analyzed the intron/exon distribution, and the results showed that the number of introns in the *MsSPL* genes ranged from one to ten (Figure 4b). Additionally, we found that nearly 66.7% of the *MsSPL* genes had fewer than four introns. Compared with other groups, Group I contained more introns, which ranged from eight to ten.

### 3.5. Multiple Sequence Alignment and Prediction of the miR156 Target Site of MsSPLs

The detailed domain structures of all MsSPLs and five previously reported protein sequences were determined. As a result, the specific SBP domain is shared by the MsSPL members (Figure S3a). The *MsSPL* genes shared 76 highly conserved amino acid residues, including two Zn finger-like structures and one NLS. Compared with C3H, C2HC was more conserved in all MsSPLs.

The previous study has isolated a miR156 precursor from alfalfa by PCR (MsmiR156d: CACGAGTGAGAGAAGACAGT). BLAST search results showed that the isolated sequence was similar to miR156d precursor in *M. truncatula* [16]. Then we compared the sequence of miRNA156 among different species, the result solidly confirms that plant miRNA156 are evolutionarily conserved Thus, ten mtr-miR156 (mtr-miR156a-j) genes were obtained from the *M. truncatula* genome to predicate miR156 target site of *MsSPLs*. Furthermore, a multiple sequence alignment was performed, which produced five types of mature miRNA sequences as shown in Figure S3b. A comparison of the miR156 mature sequences to the *MsSPL* transcript sequences indicated that 16 out of 30 *MsSPL* are complementary to the miR156 mature sequences, with a maximum of one to three mismatches which suggests that miR156 may specifically target these genes in alfalfa. Among 16 *MsSPL* genes specifically targeted by miR156, 15 *MsSPL* genes contained target sites in their coding regions, and *MsSPL08* contained a miR156 target site in the 3′-UTR. *MsSPL18* has two miR156 target sites. The miR156-targeted *SPL* genes from 11 target *OsSPL* and 10 target *AtSPL* genes were distributed into six out of eight groups (I-VIII), and most of them clustered into Group V.

### 3.6. Chromosomal Distributions, Synteny, and Evolutionary Analyses of the MsSPL

**Figure 5:**
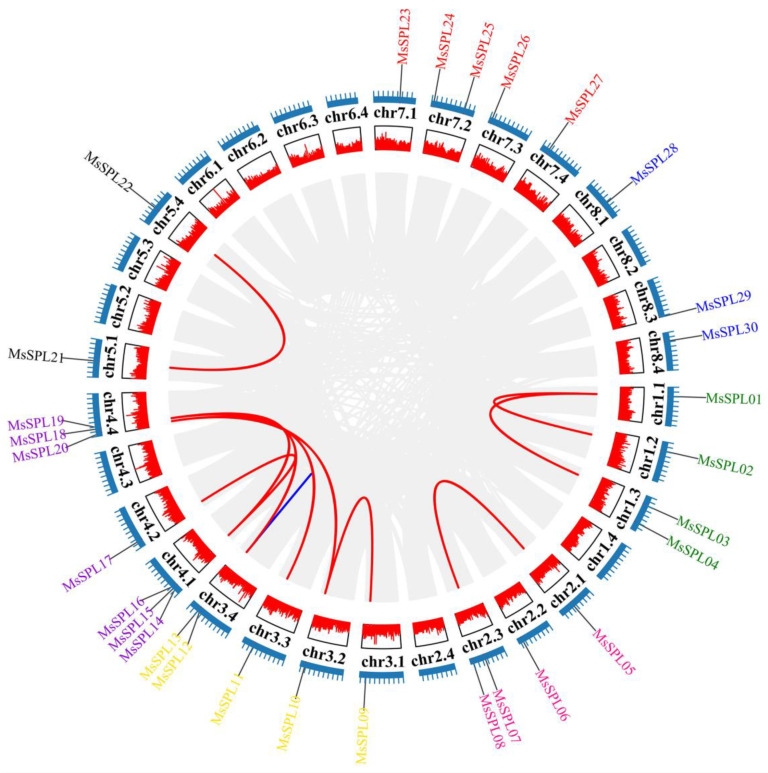
Chromosome distribution and collinearity investigation of *MsSPL* genes in alfalfa. Gray background indicates all syntenic blocks, red and blue lines indicate segmental and tandem duplications, respectively.

The physical positions of 30 *MsSPL* genes were mapped onto 22 allelic chromosomes, which represented an unbalanced distribution (Figure 5). Chromosome 4 contained the largest number of *MsSPL* genes (7; ~23.33%), whereas no gene was distributed on chromosome 6. Recently, a study showed that a round of whole-genome duplication (WGD) events were estimated to occur approximately 58 Myr ago in the alfalfa genome [6]. Among the 30 *MsSPL* genes, one gene pair of *MsSPL12/MsSPL13* displayed tandem duplications and 14 segmentally duplicated pairs located on duplicated segments on 5 chromosomes (Figure 5).

**Figure 6:**
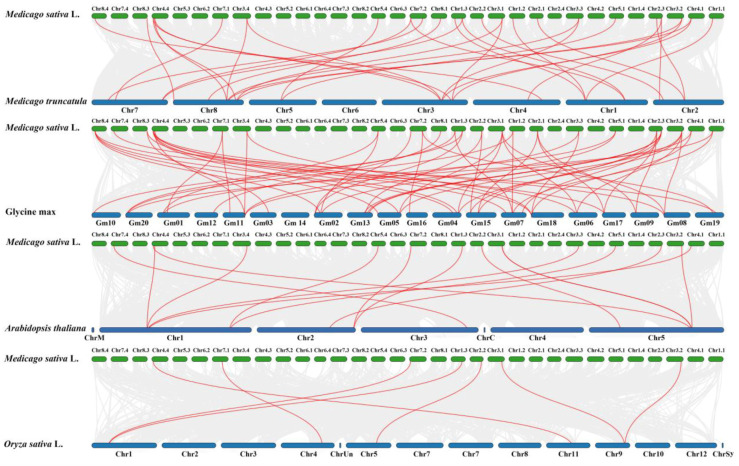
Synteny analyses *MsSPL* genes with four representative plant species. Red lines highlight syntenic *SPL* gene pairs within alfalfa and other plant genomes.

To explore the evolutionary clues for the *MsSPL* genes, three dicot plants (*M. truncatula*, *A. thaliana,* and *G. max*) and one monocot plant (*O.sativa*) were used to construct four comparative syntenic graphs (Figure 6). In total, 24 *MsSPL* gene members showed syntenic relationships with those in *G. max* (24), *M. truncatula* (23), *A. thaliana* (15), and *O.sativa* (7). The numbers of *MsSPL* orthologous genes in *G. max*, *M. truncatula*, *A. thaliana*, and *O.sativa* were 33, 17, 8, and 5, respectively. Compared to monocots, *MsSPL* consisted of more syntenic gene pairs with dicots. Furthermore, as shown in the interactive Venn diagram of *MsSPL* throughout the different species, *MsSPL04*, *MsSPL06*, *MsSPL09*, *MsSPL10*, *MsSPL20,* and *MsSPL24* had syntenic *SPL* gene pairs in all four species (Figure S4). These results indicate that the *MsSPL* genes were highly conserved and that the *MsSPL* genes were closer to the legume than to other plants. Additionally, some orthologous gene pairs were specifically mapped between alfalfa and legumes plants, including *MsSPL01*, *MsSPL02*, *MsSPL03*, *MsSPL05*, *MsSPL07*, *MsSPL25*, *MsSPL28*, *MsSPL29*, and *MsSPL30*, which may show that these orthologous pairs formed after the divergence of legume plants. These results indicated that *MsSPL* genes were closer to the legume than to others.

Furthermore, the *Ka*, *Ks*, *Ka*/*Ks,* and duplication event data of the orthologous gene pairs were estimated through Nei and Gojobori’s method (Table S3). All *MsSPL* orthologous gene pairs had Ka/Ks < 0.8, which suggests that the *MsSPL* genes might have experienced strong purifying selective pressure during evolution. The latest duplication event occurred between *MsSPL12*/*13*, which might occur ~0.11 million years ago, and all duplication events occurred after WGD events.

### 3.7. Cis-element Analysis of the Promoter

**Figure 7:**
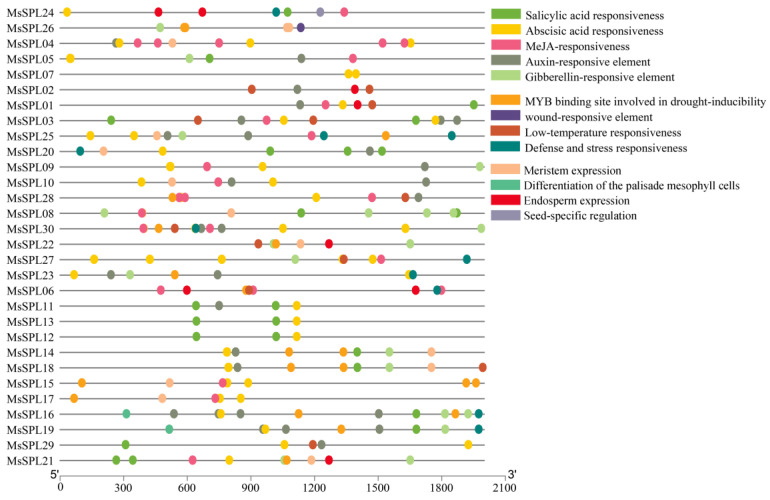
The *cis*-elements identify. The 2000 bp promoter sequences of *MsSPL* were analyzed by PlantCARE.

Previous studies have shown that *SPL* is involved in plant growth processes and abiotic stress responses. To further forecast the potential regulatory mechanisms of *MsSPL*, five hormone-related, four abiotic stress-related, and four tissue development-related elements were discovered and are displayed in Figure 7. The numbers in *MsSPL04* and *MsSPL30* are the greatest, and have the most *cis*-elements (16). *MsSPL07* contains only two abscisic acid responsiveness elements. Notably, all of the *MsSPL* genes contained at least one plant hormone-responsive element, including 27 (90%), 18 (60%), 16 (53%), 16 (53%) and 14 (47%) *MsSPL*s that had one or more abscisic acid responsive, auxin responsive, salicylic acid responsive and gibberellin responsive elements. The hormone-related elements were broadly distributed, which indicates that these genes may be regulated by phytohormones. Additionally, abiotic stress-related elements were enriched in the promoter region, and 47% and 33% of the members contained at least one MYB binding site involved in drought-induced ability (MBS) and low-temperature response (LTR) elements, which speculates that the expressions of these *MsSPLs* largely participates in signal perception and abiotic stress responses. Furthermore, some plant growth and development-related elements, including the differentiation of palisade mesophyll cells, seed-specific regulation, meristem expression, and endosperm expression elements, were also identified. The expression of *MsSPLs* with different *cis*-elements illustrates that *MsSPL* may play vital roles in regulating alfalfa growth and stress response.

### 3.8. Transcriptome and qRT-PCR Analysis Revealed Diverse Expression Patterns of MsSPL

**Figure 8:**
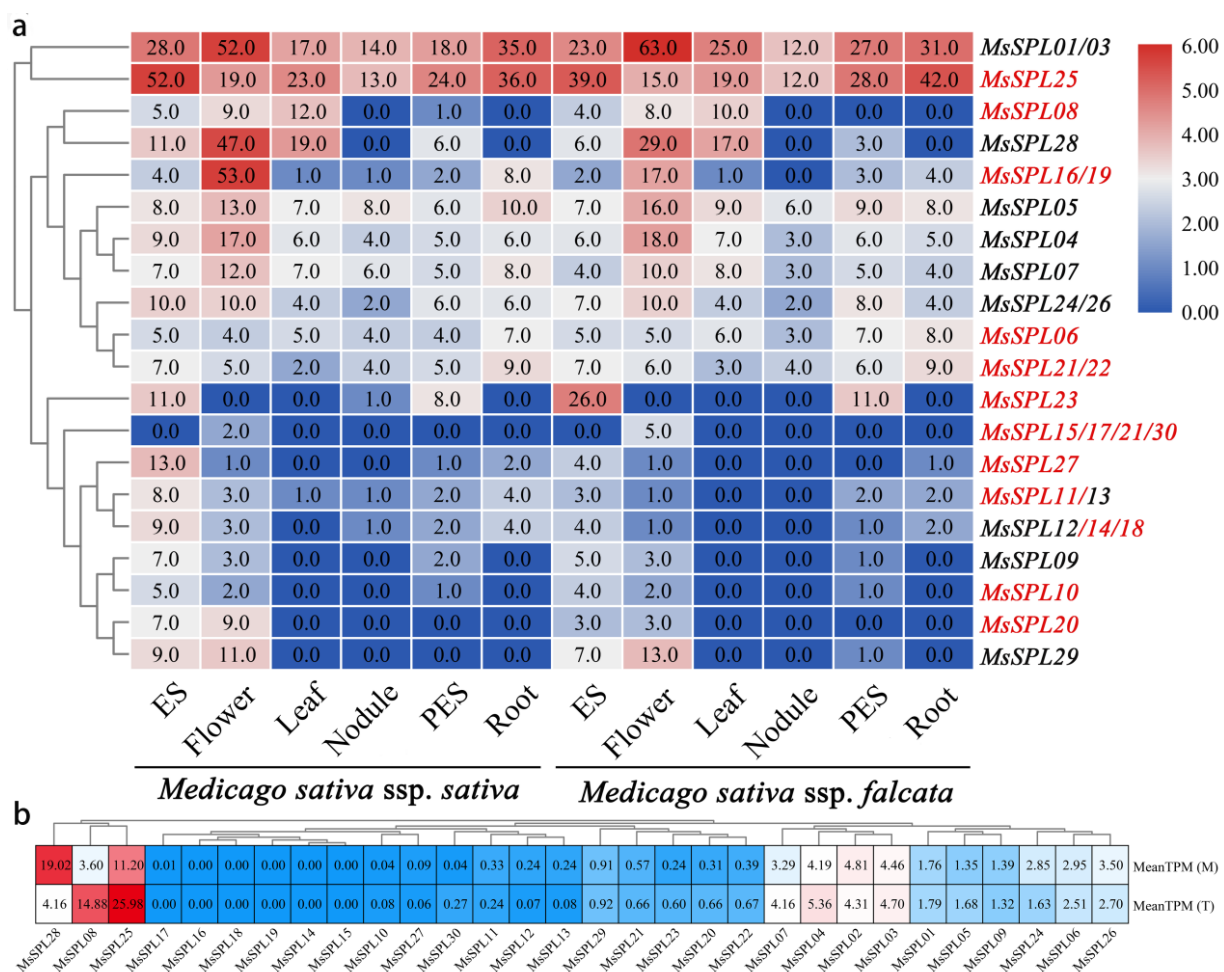
The expression pattern of *MsSPL* genes in various alfalfa tissues. (**a**) Heat map showing the expression change in PES (post-elongation stem internodes), flowers, leaf, nodule, ES (elongating stem internodes), and root. (**b**) The expression pattern of *MsSPL* genes between multifoliate and trifoliate alfalfa.

Expression profiles were mined from a comprehensive profiling study of different alfalfa tissues [30]. As shown in Figure 8a, all *MsSPLs* were expressed in at least one tissue, and nearly half of them exhibited tissue specificity. According to expression profiles of *MsSPL* members, which were divided into two groups. The first group were widely expressed across six tissues, with expression levels ranging from 0 to 63, except for *MsSPL08*, *MsSPL16*, and *MsSPL19* were not detected in roots and nodules. The second group showed relatively low expression levels in most tissues, which were highly induced in a specific tissue; for example, most *MsSPLs* were expressed in flowers and elongating stem internodes, but not detected in leaf, nodule and root tissues, including *MsSPL09*, *MsSPL10*, *MsSPL20* and *MsSPL29.* To explore the functions of *MsSPLs* in the regulation of leaflet development, a compare transcriptome between multifoliate and trifoliate alfalfa were conducted as shown in Figure 8b. Most of *MsSPLs* were not, or only lightly induced in multifoliate alfalfa, but the expression levels of *MsSPL08* and *MsSPL25* were significantly suppressed, and *MsSPL28* was significantly induced in multifoliate alfalfa.

To verify the functions of miR156 target *MsSPL* in response to drought (Figure S5a) and salt (Figure S5b) stresses, 16 *MsSPLs* were used to examine the expression profiles under PEG and salt treatments by using qRT-PCR. All miR156 target *MsSPLs* responded to PEG and salt stresses, but the response intensities and speeds were different. The majority of genes were upregulated on the fourth day of the PEG and NaCl treatments. Under drought conditions, all genes were upregulated, except for two (*MsSPL16* and *MsSPL19*) that were downregulated on the 1st day, and *MsSPL23* was downregulated on the 2nd day. After NaCl treatment for one day, three of the *MsSPL* genes (*MsSPL08*, *MsSPL10*, and *MsSPL11*) were significantly upregulated. After NaCl treatment for two and four days, most of them were greatly upregulated.

### 3.9. Paralogous of MsSPL08 Isolation and Functional Analysis in M. truncatula

**Figure 9:**
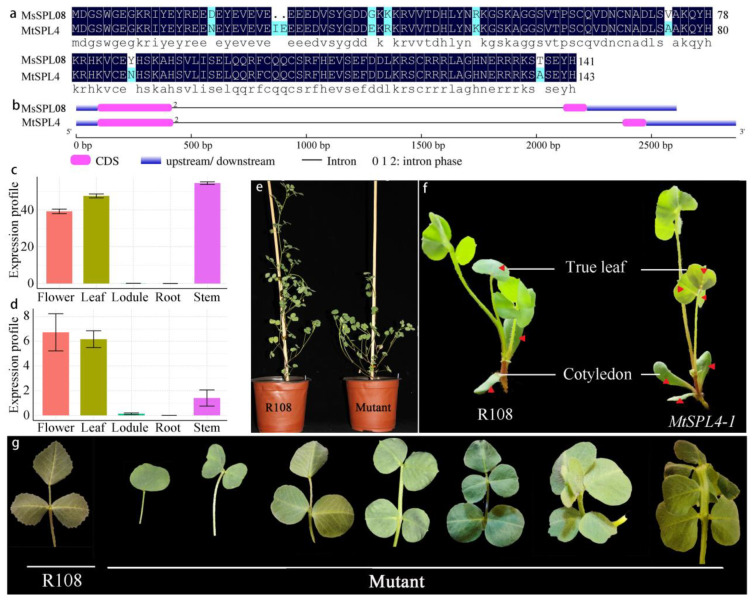
Paralogous of *MsSPL08* isolation and functional analysis. (**a**) MsSPL08 and MtSPL4 multiple sequence alignment, and (**b**) gene structure comparison. Transcript levels of *MsSPL08* (**c**) and *MtSPL4* (**d**) in alfalfa and *M. truncatula*. (**e**) The aboveground phenotype of wild type and mutant plant. (**f**,**g**) Seedling leaflet number comparison between wild type and mutant plant, the red triangle indicate the cotyledon and true leaf.

The cDNA sequences of *MsSPL08* were compared among *M. truncatula* *SPLs* (*MtSPL*) to identify the paralogous gene. A multiple sequence alignment showed that MsSPL08 has a high identity with the MtSPL4 protein sequence, which shows a 93% similarity and similar gene structure (Figure 9a,b). *M. truncatula* with a good collinearity with alfalfa, has long been considered as a model species for the studies of legume biology [32]. A significant level of sequence conservation was reported between alfalfa and *M. truncatula* allowing estimates of gene function between two species. As the sequence conservation between alfalfa and *M. truncatula* is high, we chose *M. truncatula* as a model to explore the possible functions of the miR156-targeted *MsSPL8* in compound leaf development. The expression profiles of *MsSPL08* and *MtSPL4* in different tissues showed that *MsSPL08* and *MtSPL4* play important roles in regulating aboveground tissue development (Figure 9c,d).

To determine the biological funct–ions of *MsSPL08* in compound leaf formation in alfalfa, a *Tnt1* retrotransposon-tagged *MtSPL4* mutant line was obtained. The mutant plants had more than three leaflets, increased lateral branches, a reduced plant height, and a delayed flowering time (Figure 9e), which reveals that *MtSPL4* plays important roles in compound leaf development, stem elongation, and branching. Compared with two cotyledons and one true leaf of wild-type seedlings, the mutant had three cotyledons and a trifoliate form of the true leaf (Figure 9f). The mutant had a different type of leaflet, and the number varied from one to seven, with 30.5% of the leaves exhibiting four or five leaflets in adult plants (Figure 9g).

## 4. Discussion

Alfalfa is referred to as “the queen of forage crops” because of its high biomass, biological nitrogen fixation, choice nutritional profiles, stress-tolerance and reliable sources of protein and minerals for livestock [31,33]. Increasing the leaf/stem ratio is an effective way to improve its rumen digestibility. However, the regulatory mechanism controlling leaflet formation in this species remains limited. After four years of backcross breeding, we obtained a BC_2_ population with 27.04% multifoliate rate at budding period. The single plant with the highest main branch multifoliate rate was 88%, the pinnate pentafoliate was the most stable leaflet type in BC_2_ population. To acquire more accurate information on the transcript functions, we compared the transcriptome profiles of the multifoliate (pinnate pentafoliate type) and trifoliate alfalfa genotypes. The enrichment results showed that the DEGs were mainly annotated to major metabolic and biosynthesis processes such as fructose and mannose metabolism, cysteine and methionine metabolism, ubiquinone, and another terpenoid-quinone biosynthesis, and amino acid biosynthesis.

The development of plant leaves is regulated by transcriptional regulators, plant hormones, and the mechanical properties of the tissue [34]. Among them, TFs are master regulators in response to various developmental processes and abiotic stresses in plants. Here, 227 TFs were differentially expressed, and most of them were suppressed. Previous studies have found that manipulating the expression of TF often drastically changes plant phenotypes [35]. Among many types of TF families, SPL is unique to plants [2]. The role of some *SPL* genes is post-transcriptionally regulated by miR156, as their transcripts carry functional response elements to these highly related miRNAs, which have emerged as essential regulators of various biological processes, such as phase change, leaf development, tillering/branching, stem growth, and response to stresses [36]. The chromosome-level genome assembly was completed, which offers detailed information to comprehensively analyse the *SPL* genes in this major legume forage. Through genome-wide identification, 30 *MsSPL* genes were acquired from the alfalfa genome. A phylogenetic investigation showed that the relationship of *MsSPL* is closer to *M. truncatula* and *Arabidopsis* than to rice, which suggests that eudicot *SPL* genes may diverge from a common ancestor [37,38]. Numerous *SPLs* have been identified in green plants, and the highest number of 59 *SPLs* have been recognized in *Gossypium hirsutum* and *Brassica juncea* [13], while, only 15 *SPLs* were identified in *Solanum lycopersicon* [39]. The number of *MsSPLs* is greater than that in some species such as *Citrus sinensis* [15], *Cucumis sativus* [15], *Arabidopsis* [16] and *O.sativa* [19], and close to *Populus trichocarpa* [27], *Gossypium raimondii* [29], and *M. truncatula* [22], which indicates that more *SPLs* contained in these species were attributed to WGD events. Similarly, a WGD event played a significant role in the alfalfa genome ~58 Mya ago, which allowed *SPL* gene expansion in alfalfa [6]. Motif composition and gene structure analyses indicated that *MsSPL* gene clusters in the same group shared similar motifs and exon/intron organization. Furthermore, a high divergence of motif was noticed among MsSPL proteins. For example, motifs 3 and 9 were unique to Group 1, whereas motifs 4 and 6 were only for Group 8 as compared to other SPLs, which suggests that the function of *MsSPL* genes may show diversity because of diversification [10,37,38]. In total, 16 identified MsSPLs identified have miR156 target sites, and the results showed that most of them are located in the coding region, except for MsSPL08 in the 3′-UTR. Similarly, these results were also found in *Arabidopsis*, *Hordeum vulgare*, and rice [36,40], which shows the conservation of miR156-mediated posttranscriptional regulation in different plant species [21,41,42].

In nature, leaves are an important agronomic trait, where photosynthesis converts carbon dioxide and water into carbohydrates; therefore, a high leaf/stem ratio breeding variety will increase its nutrient accumulation [43]. Leaf patterns are highly variable among different species, and even between different varieties. Among different leaf patterns and shapes, compound leaves are spectacularly diverse [44]. We analyzed the molecular mechanism by which these factors modulate leaf development to yield a substantial diversity of leaf forms in multifoliate (pinnate pentafoliate type). We found that miR156-targeted *MsSPL08* was highly expressed in leaves, stems, and flowers, but was suppressed in nodules and roots. While, compared with trifoliate alfalfa, the expression of *MsSPL08* was downregulate in multifoliate alfalfa. To further verify whether *MsSPL08* functions in leaflet differentiation, we choose *M. truncatula* as a model to carry out further research. The compound leaf composition ranged from one to seven simple leaflets, and the plant height was decreased, but the stem number was increased in *M. truncatula* mutant plants. Importantly, the mutant exhibits multifoliate traits in the seedling stage, such as three cotyledons, the true leaf is composed of three leaflets, and the serrations of the leaf edge disappear. Compared with multifoliate alfalfa, *MtSPL4* mutant plants displayed a more variable phenotype, we speculate that alfalfa is autotetraploid (2n = 4X = 32) plant, the genomes is more complex and high similarity of their subgenomes. Additionally, compound leaf development is also flexibly tuned among different species in a spatiotemporal manner. The precise regulation network of how *MsSPL08* involved in leaf patterning is also an open question.

miR156 has agronomic relevance for plant growth and stress tolerance among a wide variety of plant miRNAs. Previously studies have shown that miR156-targeted *SPL* play important role in improving biomass production and abiotic stress tolerance in legumes [36,45,46]. Similarly, we find that *MsSPL08* was also significantly induced by drought and salt stress. These observations show that the miR156/SPL module may contribute to genetic variability through the regulation of plant development and abiotic stress responses. The phenotypic changes and stress response were similar to those of the miR156-overexpressing alfalfa genotypes, including delayed flowering, reduced stem length, and increased shoot branching, and played a positive role in abiotic stress tolerance [14,47,48,49]. This observation suggests that the miR156/SPL module is integrated with the plant developmental and stress response. So far, still not clear how does miR156 integrate biological stress and regulate growth. The detailed relationship between abiotic stress response and development needs further study.

## 5. Conclusions

In this study, RNA-Seq technology was utilized to generate a comprehensive transcriptome between multifoliate and trifoliate alfalfa. Then, the RNA-Seq results were used to recognize the sequences associated with the important regulating pant trait gene *SPL*. Overall, 30 *MsSPL* were identified in alfalfa. Phylogenetic, gene structure and evolutionary analyses suggest that the functions of *MsSPL* genes may show diversity after the diversification of dicots and monocots. Based on different tissue expression profiles, *MsSPLs* can be divided into widely expressed and tissue-specific expressed groups. In this case, 16 miR156-targeted *MsSPL*s strongly implied diversification and positive roles under drought and salt conditions. Tthe transcripts of *MsSPL08* were significantly suppressed in multifoliate alfalfa. Furthermore, we used *M. truncatula* as a model to study the possible roles of miR156-targeted *MsSPL08* in regulating alfalfa compound leaf development. The phenotypes of mutant plants revealed that *miR156/MsSPL08* not only was involved in the development of multifoliate but also contributed to branches.

## Figures and Tables

**Table 1:** Data quality of RNA-Seq and mapping information.

Libraries	Raw Data		Clean Data					Mapped Reads (M)		
	Raw Reads	Raw Bases (Mbp)	Clean Reads	Clean Bases (Mbp)	Average Read Length (bp)	Q30 Bases Ratio (%)	GC Count (%)	Total Mapped (%)	Mutiple Mapped (%)	Uniquely Mapped (%)
T1	40,751,582	6112.74	3,9151,710	5738.75	146.58	93.78%	44.99%	90.10%	54.27%	35.83%
T2	41,100,934	6165.14	3,9622,384	5650.63	142.61	97.19%	44.17%	91.42%	57.70%	33.72%
T3	39,494,852	5924.23	3,7603,146	5377.32	143	92.58%	43.84%	93.24%	58.61%	34.63%
M1	44,693,794	6704.07	4,2885,164	6120.67	142.72	92.96%	44.52%	91.86%	59.18%	32.68%
M2	43,172,140	6475.82	4,1189,956	5880.61	142.77	92.70%	44.15%	94.86%	61.61%	33.25%
M3	46,502,916	6975.44	4,4872,238	6447.54	143.69	94.16%	45.09%	89.87%	57.35%	32.52%

Note: Q30 the probability that the base was miscalled is 0.1%, GC content the percentage of guanine and cytosine out of four bases. T: trifoliate, M: Multifoliate.

**Table 2:** *MsSPL* genes identified in alfalfa and their sequence characteristics.

Gene Name	Accession ID	Protein Length (aa)	Protein MW (kDa)	Protein GRAVY ^a^	*pI* ^b^	*Arabidopsis* Ortholog Locus	*M. truncatula* Ortholog Locus
*MsSPL01*	MS.gene026366.t1	1017	113.32	−0.558	7.71	ATSPL14	Medtr1g035010.1
*MsSPL02*	MS.gene040800.t1	992	110.44	−0.571	7.61	ATSPL14	Medtr1g035010.1
*MsSPL03*	MS.gene061710.t1	882	97.83	−0.564	7.52	ATSPL14	Medtr1g035010.1
*MsSPL04*	MS.gene20896.t1	1003	111.77	−0.479	6.28	ATSPL1	Medtr1g086250.1
*MsSPL05*	MS.gene98509.t1	1025	113.06	−0.356	7.12	ATSPL12	Medtr2g046550.1
*MsSPL06*	MS.gene96180.t1	747	83.81	−0.267	6.75	ATSPL7	Medtr2g020620.1
*MsSPL07*	MS.gene062030.t1	1025	113.03	−0.359	7.28	ATSPL12	Medtr2g046550.1
*MsSPL08*	MS.gene047335.t1	141	16.58	−1.294	7.2	ATSPL3	Medtr2g014200.1
*MsSPL09*	MS.gene06231.t1	376	41.68	−0.679	7.03	ATSPL13B	Medtr3g099080.1
*MsSPL10*	MS.gene055507.t1	376	41.65	−0.655	7.09	ATSPL13B	Medtr3g099080.1
*MsSPL11*	MS.gene75620.t1	434	48.02	−0.832	8.61	ATSPL2	Medtr3g085180.1
*MsSPL12*	MS.gene77585.t1	458	50.72	−0.772	8.54	ATSPL2	Medtr3g085180.1
*MsSPL13*	MS.gene045290.t1	447	49.63	−0.828	8.62	AT SPL2	Medtr3g085180.1
*MsSPL14*	MS.gene09220.t1	342	38.34	−0.796	8.44	ATSPL11	Medtr8g080690.1
*MsSPL15*	MS.gene09219.t1	368	41.73	−0.767	7.88	AT SPL10	Medtr8g080680.1
*MsSPL16*	MS.gene09218.t1	435	48.61	−0.627	5.31	ATSPL11	Medtr8g080670.1
*MsSPL17*	MS.gene023425.t1	406	46.03	−0.768	7.35	ATSPL10	Medtr8g080680.1
*MsSPL18*	MS.gene09027.t1	728	82.29	−0.775	8.33	AT SPL2	Medtr8g080680.1
*MsSPL19*	MS.gene09028.t1	445	49.57	−0.637	5.73	ATSPL2	Medtr8g080670.1
*MsSPL20*	MS.gene030453.t1	375	41.52	−0.788	8.57	ATSPL13B	Medtr8g096780.1
*MsSPL21*	MS.gene071085.t1	437	49.48	−0.707	6.6	AtSPL6	Medtr5g046670.1
*MsSPL22*	MS.gene029630.t1	437	49.45	−0.706	6.55	AtSPL6	Medtr5g046670.1
*MsSPL23*	MS.gene36181.t1	314	34.69	−0.725	8.55	ATSPL5	Medtr7g444860.1
*MsSPL24*	MS.gene28650.t1	999	110.7	−0.393	6.08	ATSPL1	Medtr7g110320.1
*MsSPL25*	MS.gene99828.t1	366	40.84	−0.846	8.62	ATSPL13B	Medtr7g028740.1
*MsSPL26*	MS.gene072658.t1	975	107.92	−0.407	6.38	ATSPL1	Medtr7g110320.1
*MsSPL27*	MS.gene04608.t1	339	36.68	−0.707	8.92	AtSPL9	Medtr7g092930.1
*MsSPL28*	MS.gene36036.t1	180	20.58	−1.187	9.2	AT SPL4	Medtr8g463140.1
*MsSPL29*	MS.gene000259.t1	337	37.43	−0.971	8.61	AT SPL8	Medtr8g005960.1
*MsSPL30*	MS.gene063508.t1	470	53.66	−0.564	6.54	AtSPL6	Medtr4g109770.1

Note: ^a^ Grand average of hydropathy (GRAVY). ^b^ Theoretical isoelectric point (*pI*).

## Data Availability

Not applicable.

## Supplementary Materials

The following are available online at https://www.mdpi.com/article/10.3390/genes13020331/s1, Figure S1: GO enrichment analysis of the DEGs. Figure S2: The expression pattern distribution of DEGs identified as TFs between multifoliate and trifoliate alfalfa. Figure S3: Multiple alignments of the MsSPL protein sequences. (a) The highly conserved SBP-domain, including two Zn-finger-like structures (C2HC and C3H) and an NLS. (a) The alignment of miR156 sequences with the target sites in MsSPL genes. Figure S4: The venn diagram of syntenic SPL genes throughout diverse species. Figure S5: The relative expression ratios of 16 miR156 target MsSPL genes under drought (a) and salt conditions (b). * *p* < 0.05 and ** *p* < 0.01. Table S1: The qRT-PCR primers used in this study. Table S2: BLAST analysis of the non-redundant genes against public databases. Table S3: The Ka, Ks and dates of the duplication evens in the paralogous MsSPL gene pairs.
