# Supplementary material for: Molecular Characterization of the miR156/MsSPL Model in Regulating the Compound Leaf Development and Abiotic Stress Response in Alfalfa
Source: Genes (Basel). 2022 Feb 10;13(2):331. doi: 10.3390/genes13020331 (PMC8871590; doi:10.3390/genes13020331)
Supplement: Supplementary file 1 [file genes-13-00331-s001.zip › genes-1486605-supplementary.pdf]

Supplement Tables

Table S1. The qRT-PCR primers used in this study.

Gene name	Forward primer (5′–3′)	Reverse primer (5′–3′)
MsSPL08	AGATGTGAGTTATGGAGATGATGG	CTCATGAAACCTGCTACATTGCT
MsSPL10	ATCAATCACCACTTCCTTTGTC	CGAATGAAACCTGCTACACTG
MsSPL11	CAATGTAGCAGGTTCCATGATCTC	GAGAGCTGATGGATTCAATTTCAC
MsSPL14	AGCTGCAGAGAACAACTTTCAC	TGTTGCTTTCCATCACAGCG
MsSPL15	CAGCGCATTCCTCTTGCTTC	ACCCAGCTGGTGAAAACCAT
MsSPL16	TGATGAGAAGCAACAGATGAACC	AATCTTCTACACCTGCATTGATCC
MsSPL17	GATGAGAAGCAACAGATGAGTCC	TCTTCTACACCTGGATTGATACTC
MsSPL18	GAAAGTCATGCCAAATCTCCC	GTTGCTTCTCATCACTGCGT
MsSPL19	TGATGAGAAGCAACAGATGAACC	AATCTTCTACACCTGCATTGATCC
MsSPL20	TCTTCATCATCTTCAACTTCAGGG	CATGGAACCTGCTACATTGCT
MsSPL21	TTCGCTTCTGAATCTATGCC	GTTACCAGATGGAACCTGCT
MsSPL22	ATGCCGATTCATCAAAGCGT	AGTTACCAGATGGAACCTGCT
MsSPL23	GGGTGAGAAGAAAGGGAAAGG	ACAACATGGAACCTGCTACAC
MsSPL25	ATGCAGCAGGTTTCATTCAC	CCAAAGTGCAGGATTCTAGGTC
MsSPL27	CAATGTAGCAGATTTCATCAGC	GGTGGTGGAAGTGAATTTCTC
MsSPL30	CCATTTGGTGATATCAGATTCCAG	ACTGAACTTACTGCCTGTAGTG
Ms-Actin	CTAGGATCCAAAATGGCCGATGGTGAGG	GAAACTCACCACCACGAACCAG

Table S2. BLAST analysis of the non-redundant genes against public databases.

Term	Number of genes	Percentage (%)
Annotated in GO	91,338	55.48
Annotated in KEGG	88,116	53.52
Annotated in Swiss Prot	116,519	70.78
Annotated in InterProScan	129,690	78.78
Annotated in TrEMBL	162,964	98.99
Annotated in NR	163,176	99.12
Total genes	164,632	100

Table S3. The *Ka*, *Ks* and dates of the duplication evens in the paralogous *MsSPL* gene pairs.

Duplicated gene pairs	Duplication type	*Ka*	*Ks*	*Ka*/*Ks*	Types of selection	Date (million year ago)
MsSPL01-MsSPL02	Segmental	0.002	0.013	0.164	Purify	0.444
MsSPL01-MsSPL03	Segmental	0.009	0.022	0.401	Purify	0.733
MsSPL02-MsSPL03	Segmental	0.008	0.012	0.684	Purify	0.402
MsSPL07-MsSPL05	Segmental	0.010	0.051	0.191	Purify	1.706
MsSPL10-MsSPL20	Segmental	0.211	0.696	0.304	Purify	23.191
MsSPL10-MsSPL09	Segmental	0.020	0.033	0.603	Purify	1.086
MsSPL11-MsSPL16	Segmental	0.330	0.793	0.415	Purify	26.445
MsSPL11-MsSPL19	Segmental	0.329	0.837	0.394	Purify	27.888
MsSPL12-MsSPL16	Segmental	0.324	0.810	0.400	Purify	27.015
MsSPL12-MsSPL19	Segmental	0.324	0.855	0.379	Purify	28.494
MsSPL16-MsSPL19	Segmental	0.011	0.014	0.766	Purify	0.473
MsSPL18-MsSPL14	Segmental	0.008	0.013	0.560	Purify	0.448
MsSPL20-MsSPL09	Segmental	0.234	0.685	0.341	Purify	22.825
MsSPL21-MsSPL22	Segmental	0.012	0.063	0.186	Purify	2.105
MsSPL12-MsSPL13	Tandem	0.002	0.003	0.583	Purify	0.110

Supplement figures

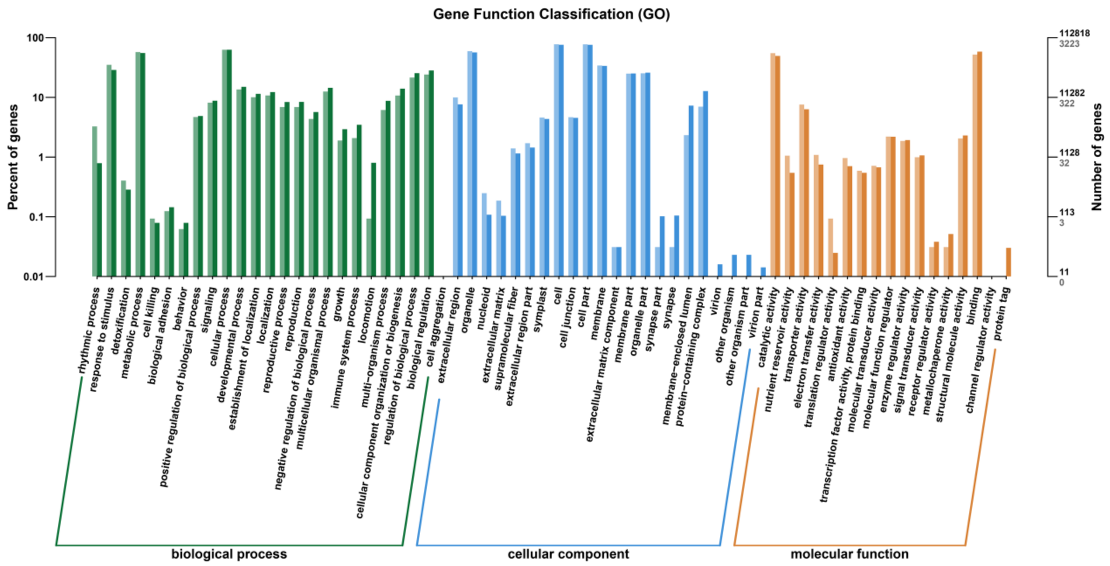

Figure S1. GO enrichment analysis of the DEGs.

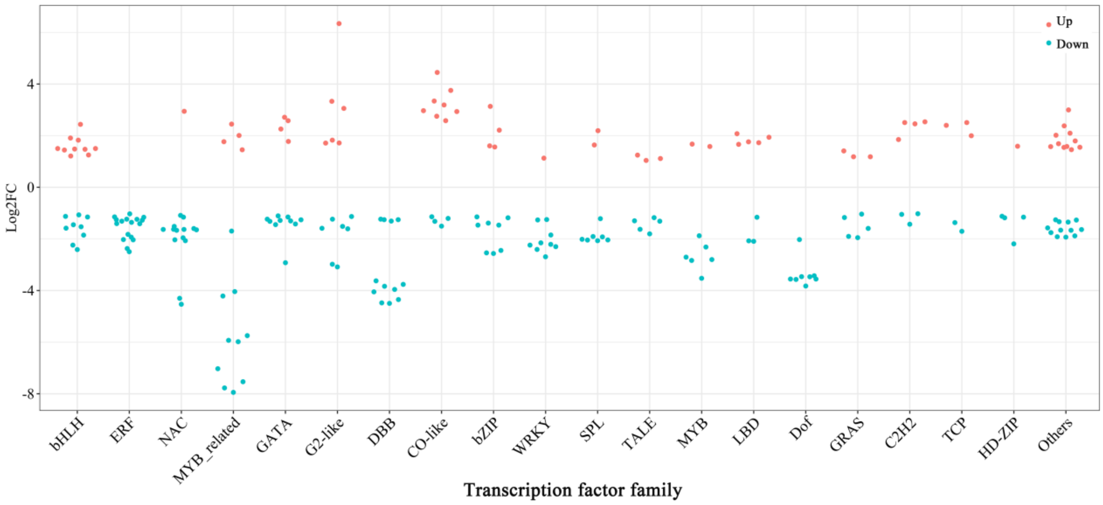

Figure S2. The expression pattern distribution of DEGs identified as TFs between multifoliate and trifoliate alfalfa.

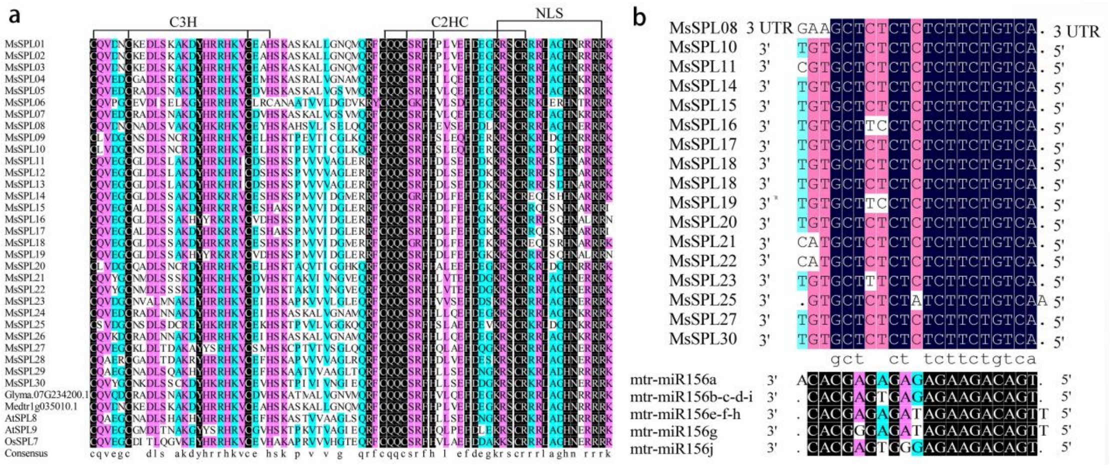

Figure S3. Multiple alignments of the MsSPL protein sequences. **a** The highly conserved SBP-domain, including two Zn-finger-like structures (C2HC and C3H) and an NLS. **b** The alignment of miR156 sequences with the target sites in *MsSPL* genes.

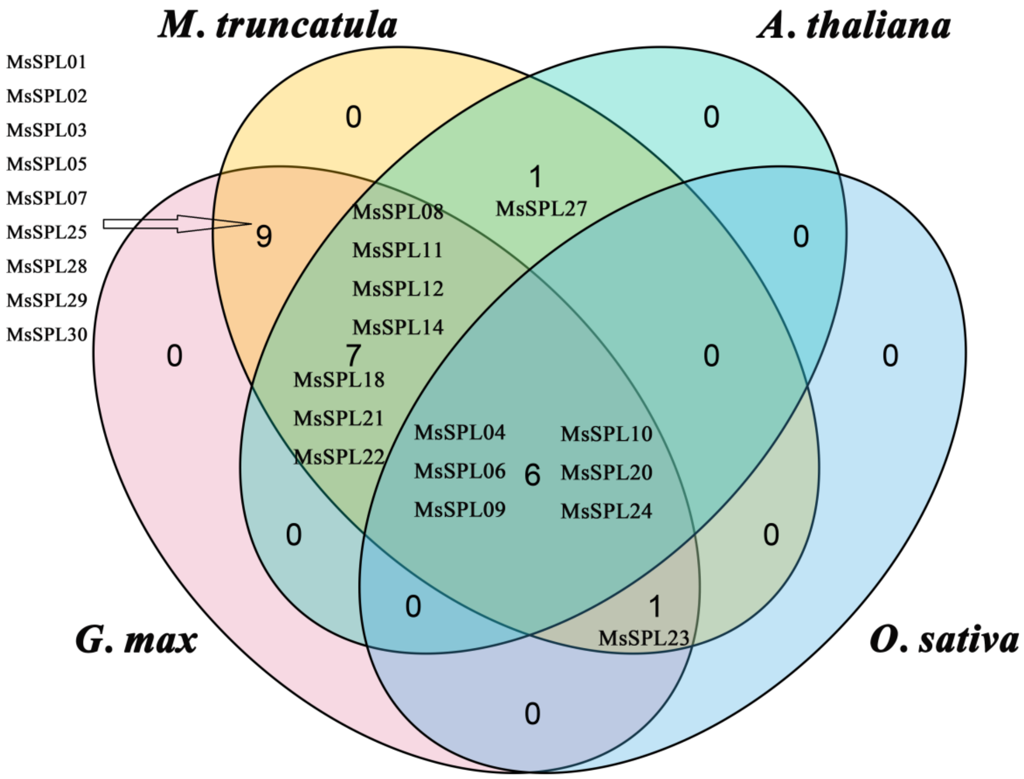

Figure S4. The venn diagram of syntenic *SPL* genes throughout diverse

species.

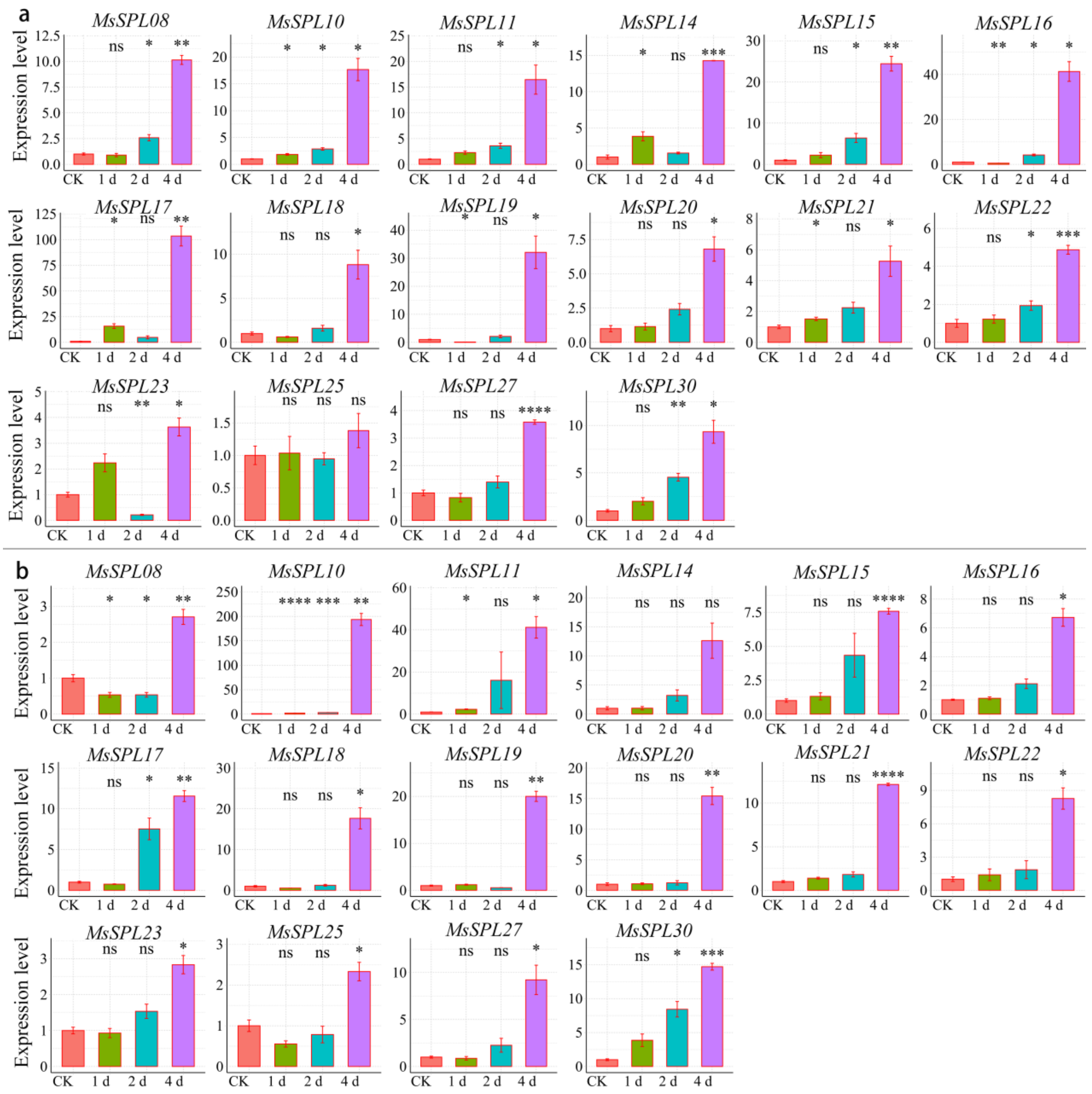

Figure S5. The relative expression ratios of 16 miR156 target *MsSPL* genes under drought (**a**) and salt conditions (**b**). *$P < 0.05$ and **$P < 0.01$.
